# Analysis of perioperative chemotherapy-mediated genomic changes in gastric cancer

**DOI:** 10.1186/s12876-025-04425-6

**Published:** 2025-11-25

**Authors:** Ko Ikegame, Hayato Omori, Masao Hada, Hideki Watanabe, Atsushi Takano, Ayako Kimura, Masayuki Inoue, Kazushige Furuya, Michiya Yasutome, Yuji Iimuro, Hiroshi Nakagomi, Kenji Amemiya, Yosuke Hirotsu, Hitoshi Mochizuki, Masao Omata

**Affiliations:** 1 Department of Surgery, Yamanashi Central Hospital, 1-1-1 Fujimi, Kofu, Yamanashi, Yamanashi 400-8506 Japan; 2 Genome Analysis Center, Yamanashi Central Hospital, 1-1-1 Fujimi, Kofu, Yamanashi 400-8506 Japan; 3 The University of Tokyo, 7-3-1 Hongo, Bunkyo-ku, Tokyo, Japan

**Keywords:** Gastric cancer, Perioperative chemotherapy, Genomic analysis

## Abstract

**Background:**

Surgery remains the mainstay of treatment for advanced gastric cancer, but in recent years perioperative chemotherapy has been administered in an attempt to improve treatment results. The Cancer Genome Atlas (TCGA) has illuminated the molecular landscape of gastric cancer. However, genomic changes before and after perioperative chemotherapy and the associated effects on treatment resistance remain unclear. This study aimed to clarify genomic change in gastric cancers treated with perioperative chemotherapy.

**Methods:**

Of the 532 patients who underwent gastrectomy for gastric cancer between January 2015 and December 2020, this study included eight patients who received neoadjuvant chemotherapy (NAC). We collected biopsy samples before NAC and surgical samples after NAC. Recurrent tumor biopsy samples after adjuvant chemotherapy were also collected in two cases. DNA and RNA were extracted from these samples and analyzed by next-generation sequencing.

**Results:**

Most of the oncogenic mutations found before NAC (*TP53*, *CDH1*, *KRAS*, *PIK3CA*, *RNF43*, and *SMAD4*) were also found in the post-NAC surgical sample. Several gene mutations with low allele frequency were lost or gained. In the recurrent biopsy samples, gene mutations shared before NAC and after NAC were also detected. In addition, some gene mutations were acquired as new mutations following surgery. Gene expression analysis showed genes related to the MAPK signaling pathway were overexpressed in the group without recurrence.

**Conclusions:**

Most of the oncogenic mutations were maintained throughout perioperative chemotherapy and remained in recurrent tumors. The development of drugs that affect oncogenic mutations during perioperative chemotherapy is required.

## Introduction

Gastric cancer cases and deaths are on the decline worldwide, but incidence and mortality rates are still high in Asia [1]. Recent advances in chemotherapy for advanced gastric cancer have resulted in prolonged survival, but the results are still unsatisfactory. Surgery is the mainstay of treatment for advanced gastric cancer, but various perioperative chemotherapy regimens have been tried to improve outcomes [2]. Postoperative adjuvant chemotherapy is the standard of care in Japan, while preoperative chemotherapy is standard in Europe and the United States [3–5]. Further perioperative chemotherapy development is expected with the advent of molecularly targeted therapies and ICIs (immune checkpoint inhibitors).

Comprehensive and large-scale genomic analysis of gastric cancer has revealed its genetic characteristics [6, 7]. The Cancer Genome Atlas (TCGA) group’s comprehensive cancer genome analysis, the primary focus of this project, has classified four subtypes and reported their characteristic genomic abnormalities. The development of new therapies is expected for each subtype [6]. TCGA study excluded patients who received preoperative chemotherapy, and the effects of perioperative chemotherapy on the tumor and its microenvironment remain unclear. Because most gastric cancer patients do not respond well to preoperative chemotherapy, the search for new molecular markers to predict response is an urgent issue. By clarifying which genomic alterations are associated with chemotherapy resistance, it may be possible to select patients who respond well to preoperative chemotherapy. The aim of this study was to identify genomic alterations across time in gastric cancer patients undergoing perioperative chemotherapy.

## Methods

### Subjects and clinical samples

Surgical samples were obtained from 532 gastric cancer patients who received gastrectomy at our hospital between January 2015 and December 2020. Of the 11 patients who received preoperative chemotherapy, we included eight patients who had preoperative endoscopic biopsy samples. Two patients had endoscopic biopsy samples for anastomotic and colon recurrence, and these samples were included in the analysis. Pathological stage was determined according to the Japanese Classification of Gastric Cancer [8]. The treatment strategy was in accordance with the Japanese Guidelines for the Treatment of Gastric Cancer [5]. The pathological evaluation was conducted according to the Japanese system of histological evaluation of tumour response. This includes: grade 0, no effect; grade 1, slight effect (1a: viable tumour cells occupy >2/3 of the tumour area, 1b: viable tumour cells remain in < 2/3 of the tumour area); grade 2, considerable effect (viable tumour cells remain in < 1/3 of the tumour area); and grade 3, complete response (no viable tumour cells remain).

### Blood and tissue sample Preparation and DNA extraction

After centrifuging peripheral blood samples, buffy coats were isolated and stored at − 80 °C until DNAextraction [9]. Buffy coat DNA was extracted using the QIAamp DNA Blood Mini QIAcube Kit (Qiagen, Hilden, Germany) with QIAcube (Qiagen). DNA concentration was determined using the NanoDrop 2000 spectrophotometer (Thermo Fisher Scientific, Waltham, MA, USA). We fixed tumor specimens with 10% buffered formalin to make formalin-fixed, paraffin-embedded (FFPE) tissues [10]. FFPE tissues were stained with hematoxylin and eosin and microdissected using the Arcturus LCM System (Thermo Fisher Scientific) to enrich tumor tissue. Subsequently, FFPE DNA was extracted using the QIAamp DNA FFPE Tissue Kit (Qiagen).

### Whole-exome sequencing (WES)

Multiplex PCR was conducted using genomic DNA from buffy coats and tumor FFPE DNA with a premixed primer pool using the Ion AmpliSeq™ Exome RDY (Thermo Fisher Scientific). PCR products were pooled and treated with FuPa reagent to partially digest primer sequences. The amplicons were ligated to adapters with the diluted barcodes of the Ion Xpress Barcode Adapters Kit (Thermo Fisher Scientific). Purification was performed using Agencourt AMPure XP reagents (Beckman Coulter, Brea, USA). Library concentrations were determined using the Ion Library Quantitation Kit (Thermo Fisher Scientific). Emulsion PCR and chip loading were performed on the Ion Chef using the Ion PI Hi-Q Chef Kit. Sequencing was performed using the Ion PI Hi-Q Sequencing Kit on the Ion Proton Sequencer (Thermo Fisher Scientific).

### RNA extraction

RNA was extracted from the dissected samples using an RNeasy FFPE Kit (QIAGEN). 10 ng of total RNA was reverse transcribed, the resulting cDNA was amplified for 20 cycles by adding PCR Master Mix and the AmpliSeq human transcriptome gene expression primer. Amplicons were digested with the proprietary FuPa enzyme, then barcoded adapters were ligated onto the target amplicons. Libraries were eluted and individually quantitated by qPCR using Ion Torrent P1 and A sequencing primers and SYBR Green master mix. Transcriptome sequencing was performed using the Ion AmpliSeq Transcriptome Gene Expression Kit (Thermo Fisher Scientific, USA).

### Bioinformatics analysis

Differential gene expression analysis was performed between the groups with recurrence and without recurrence, and significantly different genes were indicated using volcano plots. Characteristic genes with log2 foldchange > 2 and -log10(p) > 2 were extracted and annotated using Metascape (http://metascape.org/gp/index.html#/main/step1). In addition, genes associated with characteristic pathways were searched using the Kyoto Encyclopedia of Genes and Genomes (KEGG).

### Statistical analysis

All statistical analyses were conducted using R software version 4.1.2 (R Foundation for Statistical Computing, Vienna, Austria). Statistical significance was set at *p* < 0.05.

## Results

### Patient characteristics

**Fig. 1:**
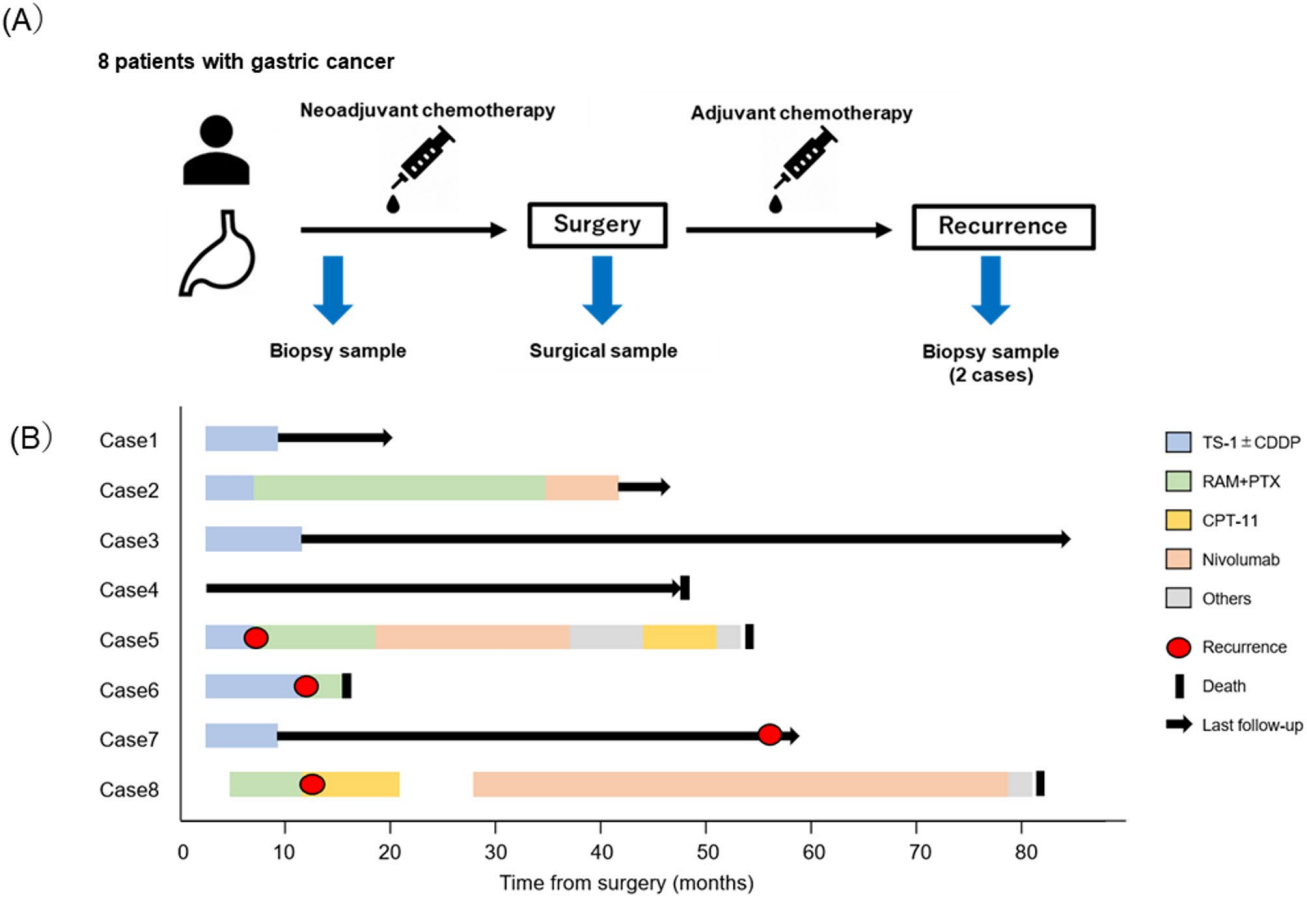
**A** The timeline of the sampling process. **B** The Swimmer’s plot of the treatment and course of treatment after surgery for each case RAM + PTX, ramucirumab plus paclitaxel; CPT-11, irinotecan

Figure 1A provides a timeline of the sampling process. Patient background characteristics are shown in Table 1. The average age was 70 years, and only Case 7 was female. Almost all patients had advanced lymph node metastasis of N2 or more. In addition, Cases 2, 7, and 8 were positive for periaortic lymph node metastasis and were cStageIVb. Preoperative chemotherapy consisted of a regimen of cisplatin or oxaliplatin in combination with TS-1. Most of the surgical pathology specimens were poorly differentiated (Table 2). The therapy effect of preoperative treatment was grade 2 or higher only in Cases 3 and 4. Radical gastrectomy was performed after preoperative chemotherapy except in Case 2 and Case 7. Figure 1B shows a Swimmer’s plot of the treatment and course of treatment after surgery for each case. Postoperative adjuvant chemotherapy including TS1 was selected except in Cases 4 and 8. Three patients were treated with nivolumab after the third line of treatment. Four deaths were observed in the course of the disease, and Case 4 was another cancer death due to hepatocellular carcinoma.

**Table 1:** Patient characteristics

Case number	Age (years)	Sex	Tumor location	Gross classification	cT	cN	cM	cStage	NAC	Surgical procedure
1	69	Male	U	2	4b	3a	0	III	S-1 + CDDP	LPG
2	76	Male	ML	2	4a	3b	1 (LYM)	IVb	SOX	ODG
3	48	Male	M	3	4b	3a	0	III	SOX	OTG
4	75	Male	M	2	4a	0	0	IIB	SOX	OPG
5	71	Male	U	3	4b	2	0	III	S-1 + CDDP	OPG
6	82	Male	L	3	4a	3a	0	III	SOX	ODG
7	71	Female	U	3	4a	3a	1 (LYM)	IVb	SOX	ODG
8	71	Male	M	3	4a	2	1 (LYM)	IVb	S-1 + CDDP	OTG

*LYM* lymph nodes, *NAC* neoadjuvant chemotherapy, *CDDP* cisplatin, *SOX* S-1 + oxaliplatin, *LPG* laparoscopic proximal gastrectomy, *ODG* open distal gastrectomy, *OPG* open proximal gastrectomy, *OTG* open total gastrectomy

**Table 2:** Pathological features and therapy effect

Case number	Histological type	Tumor size (mm)	Lymphatic invasion	Venous invasion	ypT	ypN	CY	ypStage	Therapy effect
1	Poorly differentiated	75	Positive	Positive	3	3a	0	IIIB	0
2	Poorly differentiated	180	Positive	Positive	4a	3b	1	IV	1a
3	Poorly differentiated	30			1b	0	0	I	2
4	Poorly differentiated	0			0	0	0	0	3
5	Poorly differentiated	30	Positive	Positive	3	2	0	IIIA	1b
6	Poorly differentiated	40	Positive	Positive	4a	3b	0	IIIC	0
7	Mucinous	100	Positive		4a	3a	0	IV	1a
8	Poorly differentiated	50	Positive	Positive	4a	3a	0	IIIA	1a

*CY* peritoneal cytology

### Gene mutation profiling

**Fig. 2:**
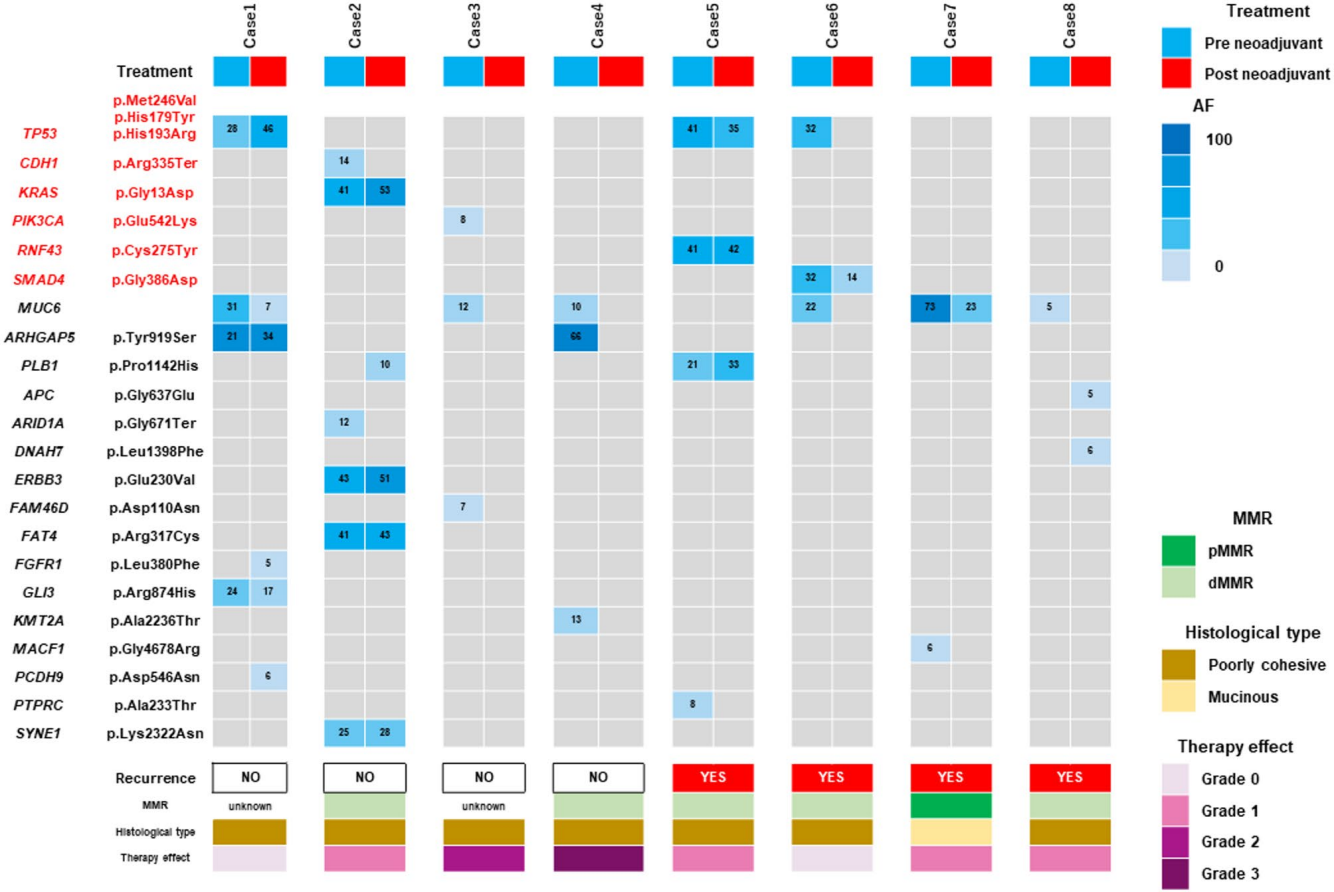
The changes in gene mutation profiles before and after preoperative chemotherapy Blue columns indicate pre-NAC and red columns indicate post-NAC results. Gene mutations marked in red are oncogenic mutations AF, allele frequency; pMMR, mismatch repair-proficient; dMMR, mismatch repair-deficient

Figure 2 shows the changes in gene mutation profiles before and after preoperative chemotherapy. Oncogenic mutations are marked in red. The most frequent mutation was *TP53*, which was found in three cases. Other oncogenic mutations were *CDH1*, *KRAS*, *PIK3CA*, *RNF43*, and *SMAD4*. Many oncogenic mutations remained mutated and alle frequency (AF) values were maintained after preoperative chemotherapy. *Tp53* in Case 6 and *PIK3CA* in Case 3 were not detected after chemotherapy. Mutations other than oncogenic mutations with an AF greater than 20 did not change after preoperative chemotherapy. Case 7 was pMMR (mismatch repair-proficient), while all other cases were dMMR (mismatch repair-deficient).

**Fig. 3:**
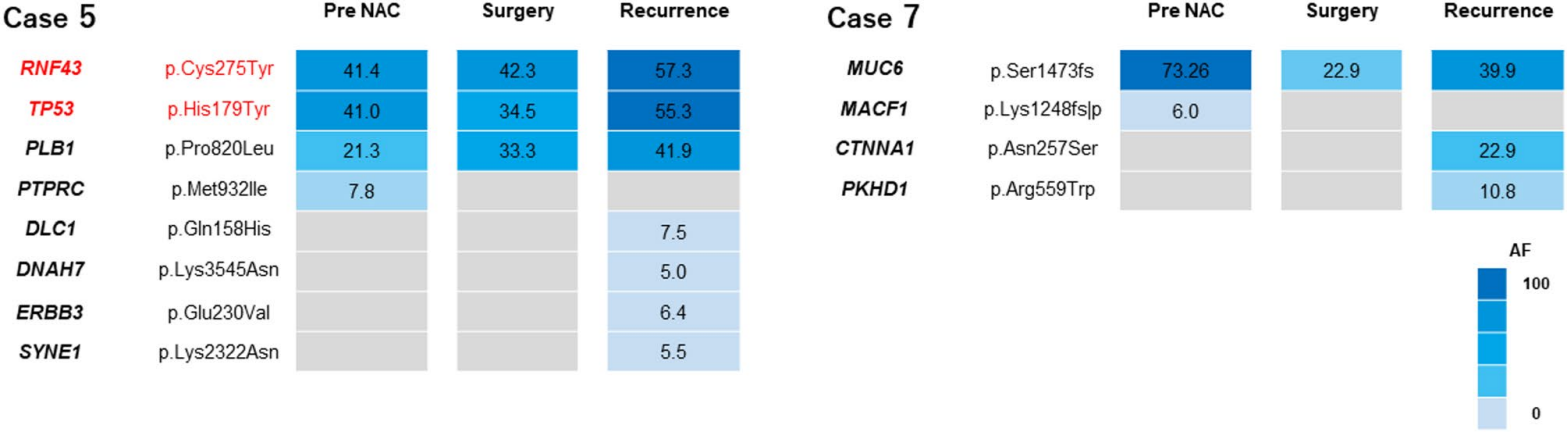
The perioperative chemotherapy-mediated genomic changes in cases with samples of recurrence Gene mutations marked in red are oncogenic mutations AF, allele frequency

Figure 3 shows perioperative chemotherapy-mediated genomic changes in two cases for which recurrent tissue samples were obtained. In Case 5, the high AF values of oncogenic mutations *RNF43* and *TP53* were maintained. Although AF was low, *DLC1*, *DNAH7*, *ERBB3*, and *SYNE1* were extracted as new-emerging mutations at recurrence. *RNF43*, *TP53*, and *PLB1* all had elevated AF in the recurrent samples. Similarly, *MUC6* mutations were maintained in Case 7. In addition, *CTNNA1* and *PKHD1* were detected as new mutations in the recurrent samples.

### Gene expression profiling

**Fig. 4:**
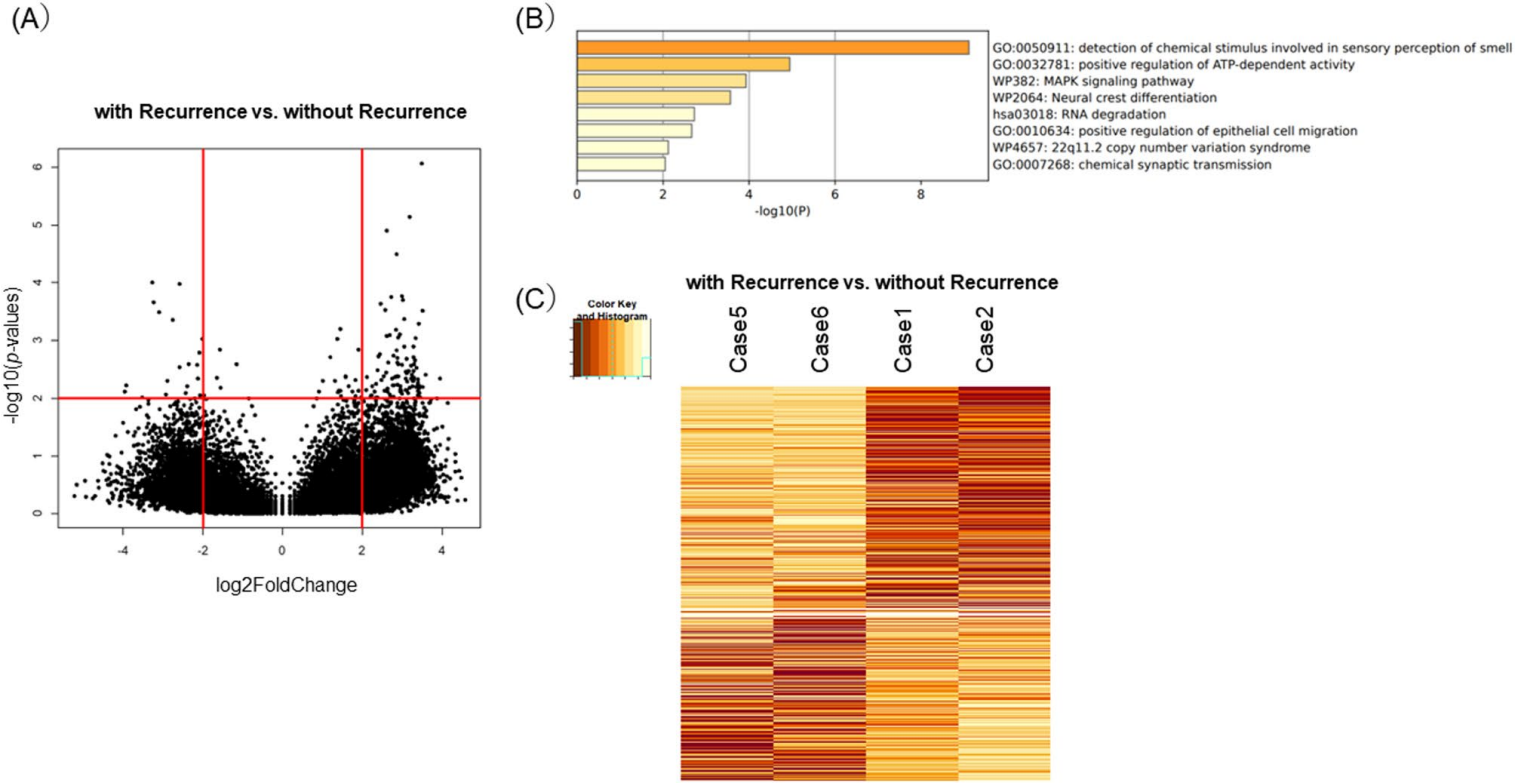
Gene expression profiling. **A** Volcano plot representing the differential gene expression of the analyzed genes in patients with recurrence and without recurrence. **B** Gene Ontology and KEGG enrichment analysis of the shared genes using metascape. **C** The heatmap of gene set related to the MAPK signaling pathway

Figure 4 shows the results of gene expression analysis of the four surgical samples (Case1, 2, 5 and 6) for which RNA libraries could be created. The differences in gene expression between the groups with recurrence and without recurrence are shown in the volcano plot (Fig. 4A). We extracted characterized lists of 20 genes in the group with recurrence and 99 genes in the group without recurrence. Enrichment analysis of the 99 genes in the non-recurrence group by Metascape extracted the characteristic pathway profile of the MAPK signaling pathway (Fig. 4B). Gene sets related to the MAPK signaling pathway were selected from the KEGG pathway database to create a set of 750 genes. A heatmap of this gene set showed that the genes overexpressed in those groups were accumulated in the group without recurrence (Fig. 4C).

## Discussion

This study shows perioperative chemotherapy-mediated genomic changes in gastric cancer. Oncogenic mutations in each tumor were maintained throughout the treatment period, and the mutations remained in recurrent tumors. This study suggests the need to develop drugs that affect oncogenic mutations in perioperative chemotherapy.

Surgery is the main treatment for resectable, advanced gastric cancer. Perioperative chemotherapy is the standard for locally advanced gastric cancer in Europe and the United States. The MAGIC trial demonstrated the benefit of this approach with the three-drug combination of epirubicin, cisplatin, and fluorouracil [3]. Furthermore, the FLOT4 trial demonstrated the usefulness of FLOT, and it has been selected as a standard treatment [4]. In Asia, the PRODIGY trial in Korea and the RESOLVE trial in China reported the usefulness of preoperative chemotherapy with DOS and SOX, respectively [11, 12]. Preoperative chemotherapy attacks potential cancer cells in patients who are likely to develop distant metastases and may provide useful information for adjuvant chemotherapy. In Japan, preoperative chemotherapy has been used for gastric cancer patients with scirrhous tumors or extensive lymph node metastasis, and this treatment should be further supported by forthcoming data from clinical trials [5, 13].

In most previous studies of gastric cancer genomes, tissues were analyzed at a single point due to the difficulty of collecting samples intermittently over a long period. This study is useful because samples were collected over time at a single site with correlated clinical information. Anastomotic recurrence in Case 5 and colon recurrence in Case 7 were analyzed. Both samples were pathologically determined to be tumor tissues similar to the primary tumor and were diagnosed as metastases. In each case, major genetic mutations were maintained, supporting the diagnosis of recurrence. Ziyu et al. compared samples before and after preoperative chemotherapy and identified *C10orf71* and *IRS1 as* mutations associated with treatment resistance [14]. No cases in our cohort showed these mutations, making it difficult to assess these claims. Yeon et al. also reported the results of a genomic analysis comparing surgical samples and recurrence samples [15]. Similar to our report, the oncogenic mutations did not disappear but remained in the recurrent lesions, and AF tended to be increased in the recurrent samples. Our results also suggest that mutations with elevated AF and new-emerging mutations in recurrent samples are associated with treatment resistance. However, as in Case 6, there were instances where oncogenic mutations such as Tp53 were reduced despite the low therapeutic effect. This may suggest that the pathological and genetic effects of treatment may not be correlated. The MAPK signaling pathway regulates multiple critical cellular functions including proliferation, growth and senescence of normal cells [16]. In recent years, mutations in the MAPK signaling pathway have been implicated in tumor promotion processes, and attention has focused on developing drugs that inhibit these pathways. Genes related to the MAPK signaling pathway were overexpressed in the group without recurrence in this study. Because of the limited number of cases in this cohort, further studies on the association between the MAPK pathway and treatment resistance are needed.

In another study at our institution, we reported genomic alterations before and after preoperative chemotherapy, including trastuzumab, for HER2-positive breast cancer [17–20]. In the non-CR group, there was an increase in the frequency of *TP53* mutations in patients with recurrence, but none of the patients without recurrence showed these changes. Even if preoperative chemotherapy does not achieve CR, chemotherapy that can affect oncogenic mutations are assumed to produce better outcomes. Gastric cancer has a high frequency of heterogeneity in molecular biology, which makes it difficult to develop effective ICI therapies [21]. And it is estimated to show even more variety of changes before and after chemotherapy. Theoretically, ICIs may be useful in preoperative chemotherapy, because treatment is initiated in the absence of damage to the immune system [22]. Future development of preoperative chemotherapy including ICI therapy in gastric cancer is expected.

Limitations of this study are as follows: It was a single-center, retrospective analysis. Preoperative chemotherapy was not the standard treatment in Japan, and the analysis was conducted in a small cohort. We look forward to accumulating more cases to increase the reliability and validity of the study. RNA libraries were difficult to prepare for all samples, and the formalin fixation of the tumor tissue is assumed to be the cause of poor RNA extraction due to aging deterioration.

## Conclusion

In conclusion, we report the results of an analysis of perioperative chemotherapy-mediated genomic changes in gastric cancer, showing that oncogenic mutations were maintained throughout perioperative chemotherapy and remained in recurrent tumors. The development of drugs that affect oncogenic mutations during perioperative chemotherapy is required.

## Data Availability

Data analyzed in this study are available from the corresponding author, upon reasonable request.
